# Immune activation of murine RAW264.7 macrophages by sonicated and alkalized paramylon from *Euglena gracilis*

**DOI:** 10.1186/s12866-020-01782-y

**Published:** 2020-06-19

**Authors:** Qingqing Guo, Decheng Bi, Mingcan Wu, Boming Yu, Lang Hu, Chenchen Liu, Liang Gu, Hui Zhu, Anping Lei, Xu Xu, Jiangxin Wang

**Affiliations:** 1 Shenzhen Key Laboratory of Marine Bioresources and Eco-environmental Science, Shenzhen Engineering Laboratory for Marine Algal Biotechnology, Guangdong Provincial Key Laboratory for Plant Epigenetics, College of Life Sciences and Oceanography, Shenzhen University, Shenzhen, 518060 China; 2 College of Food Engineering and Biotechnology, Hanshan Normal University, Chaozhou, 521041 China; 3 College of Physics and Optoelectronic Engineering, Shenzhen University, Shenzhen, 518060 China

**Keywords:** Paramylon, *Euglena gracilis*, Immune activation, Nuclear factor-κB, Mitogen-activated protein kinase, Macrophages

## Abstract

**Background:**

*Euglena* is a new super health food resource that is rich in the natural polysaccharide paramylon, a linear β-1,3-glucan with various biological activities including activity on the immune system in different cell lines and animals. Despite these reports, the immune regulation mechanism of paramylon is still unclear.

**Results:**

We investigate the signaling pathways paramylon impacts in immune macrophages. In RAW264.7 macrophages, sonicated and alkalized paramylon oligomers up-regulated inducible nitric oxide synthase (iNOS) and increased secretion of nitric oxide (NO), interleukin (IL)-6 and tumor necrosis factor (TNF)-α, in a concentration-dependent manner. In addition, paramylon activated the nuclear factor-κB(NF-κB) and mitogen-activated protein kinase (MAPK) signaling pathways and inhibiting these pathways attenuated the paramylon-induced secretion of the above immune-mediators.

**Conclusions:**

These results demonstrate that *Euglena gracilis* paramylon modulates the immune system via activation of the NF-κB and MAPK signaling pathways and thus has potential therapeutic benefits.

## Background

The unicellular flagellate model eukaryote *Euglena gracilis* produce the storage polysaccharide paramylon which consist of a linear β-1,3-glucan chain. Paramylon is essentially different from other β-glucans normally integrated in the cell walls such as in the cell walls of yeast and fungi, as it is stored in rod like bodies throughout the cytoplasm of *Euglena* [1–3]. Under optimal culture conditions, paramylon content can reach 50–70% of dried biomass in some *Euglena* species. As they can be produced on an industrial scale in microorganisms, paramylon and other high molecular weight β-1,3-glucans are also ideal nutritional supplements.

Among potential immune drugs are the β-glucans, which are a group of polysaccharide occurring in all branches of the tree of life including plants, algae, bacteria and fungi [4]. β-glucans form heterogeneous polysaccharide groups, and β-glucans have immune activity depending on their molecular structure, including size, branching frequency and conformation [4]. These β-1,3-glucans have various biological activities in mammals, including protecting against cholesterol, diabetes, hypoglycemia, inflammation, liver disease, tumors, microbial and viruses infections [1, 5]. Quesada et al. reported that intraperitoneal injection of paramylon at 24 h post tumor transplantation has an inhibitory effect on tumor growth, although it did not cause complete tumor regression [6]. Watanabe et al. found that paramylon significantly inhibited pre-neoplastic aberrant crypt foci development in the colon of mice, and that the paramylon had a preventive effect on colon cancer [7]. In hemocytes of bivalves, exposure to β-glucans increases nitric oxide production, peroxidase and antibacterial activity, and phagocytosis both in vitro and in injection-based experiments [8–11]. It was previously reported that paramylon stimulated tumor TNFα in murine J774 macrophage cells, although the mechanisms were not further investigated [12]. In addition, we recently demonstrated that a sea-weed β-glucan, BG136, can activate the murine macrophage cell line RAW264.7 by binding TLR4 to trigger cytokine secretion, including the activation of the MAPK and NF-κB signaling pathways [13]. We thus hypothesized that paramylon might also stimulate these pathways.

Mammalian immunity comprises the innate and adaptive immune systems. The innate immune system is the first line of defense against host microbial infections and is mediated by phagocytic cells including macrophages and neutrophils [14]. At rest, macrophages have only basic phagocytic and proliferative functions [15]. However, once the body is stimulated by foreign bodies, macrophages are activated, causing them to produce various inflammatory mediators, such as interleukin (IL), interferon (IFN), tumor necrosis factor (TNF), nitric oxide (NO) [8] and reactive oxygen species (ROS) [16]. These inflammatory factors can also feedback to regulate or activate the immune cells, which then phagocytoze and neutralize the inflammatory factors to restore the health of cells and tissues [17].

The immune system is activated by various signaling pathways, notably the Nuclear factor-κB (NF-κB) and mitogen-activated protein kinase (MAPK) signaling pathways. NF-κB plays a key role in the innate immune response by regulating multiple immune-response genes [18]. After stimulation, the cells activate the NF-κB dimer and separate it from the IκB inhibitor. The activated NF-κB dimer enters the nucleus and regulates the expression of inflammation mediators, thereby participating in the inflammatory response. Another important signaling pathway, the MAPK pathway, also regulates the expression of inflammation mediators in the innate immune response, through protein phosphorylation [19]. Clearly the NF-κB and MAPK pathways are valuable potential therapeutic targets. However, other pathways of the immune system may be also sensitive to β-glucans, such as those regulated by Toll-like receptors (TLRs), RIG-I-like receptors (RLRs), Nod-like receptors (NLRs) and AIM-2-like receptors (ALRs), C-type lectin receptors (CLRs) and other DNA sensors [20].

Although the immunological activity of β-glucans, like paramylon, has been studied, the molecular mechanisms behind such as regulation mechanism and signaling pathways involved are largely unknown.

## Results

### Paramylon triggers NO release

Paramylon is one of several promising immune reagents, however, its activity has mostly been investigated in cell lines or mammals [12, 21]. In this study we therefore sought to purify paramylon from *E. gracilis* and test its biological activity. First, we prepared paramylon by sonication and alkaline treatment to reduce the degree of polymerization. We then ran a battery of tests on our purified preparation.

**Fig. 1:**
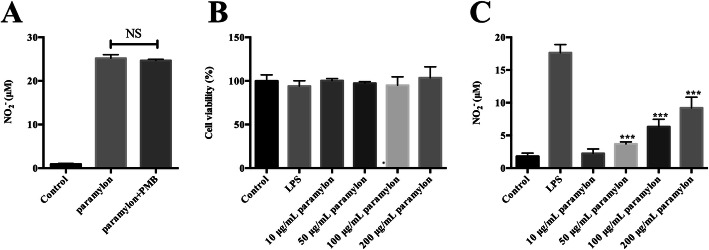
Paramylon triggers NO release in RAW264.7 macrophages. **a** After stimulation of macrophages with paramylon (10, 50, 100, and 200 μg/mL), in the presence or absence of the lipopolysaccharide-inhibitor polymyxin B (PMB), the NO production in the culture supernatant was measured with Griess reagent. **b** Cell viability, was measured with a cell counting kit. **c** Paramylon dose responses. NO production in the culture supernatant was measured using Griess reagent as in (**a**). Representative images and results from three independent experiments are shown. NS, not significant, *** P < 0.001

Secondly, we tested NO release in response to paramylon in macrophages. We treated RAW264.7 macrophages with 200 μg/mL paramylon, with or without the lipopolysaccharide inhibitor polymyxin B (2.5 μg/mL), and measured NO release in the medium 24 h later, with a SpectraMax microplate reader. NO was released at medium and there was no impact of polymyxin B (Fig. 1a).

Endotoxin lipopolysaccharide is a potent inducer of inflammatory mediators [22, 23]. Therefore, the lack of effect of polymyxin B proves that our paramylon preparation is free from endotoxin contamination. Importantly also, neither paramylon nor lipopolysaccharide affected cell viability, up to 200 μg/mL (Fig. 1b). After treatment with 100 ng/mL lipopolysaccharide and 10, 50, 100, 200 μg/mL of paramylon for 24 h in the medium, the nitrite level was measured to evaluate NO production. As shown in Fig. 1c, NO release was induced by paramylon in a dose-dependent manner in RAW264.7 macrophages.

### Paramylon enhances iNOS levels

**Fig. 2:**
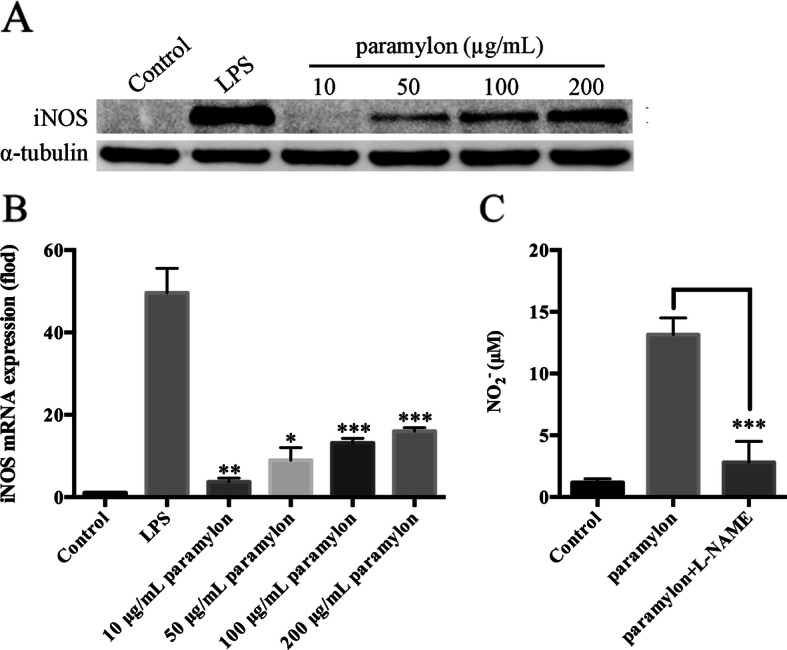
Paramylon induces iNOS expression leading to NO release. **a** After incubation of RAW264.7 cells with paramlyon for 24 h, the level of iNOS protein was examined by Western blot. **b** iNOS mRNA levels were also analyzed after 12 h of incubation by qRT-PCR. **c** The level of NO in the medium was measured in the presence or absence of the iNOS inhibitor NG-nitro-L-arginine methyl ester (L-NAME, 1 mM). Figures show the means and standard errors of three independent experiments are shown. * P < 0.05, ** P < 0.01, *** P < 0.001

The production of NO in the inflammatory response is mainly regulated by iNOS. Studies have shown that macrophages can produce large amounts of iNOS, which regulates the production of NO [24]. Paramylon significantly up-regulated the expression of iNOS RNA and protein in RAW264.7 cells, in a concentration-dependent manner (Fig. 2a, b). Importantly, the paramylon-triggered NO production was attenuated by the NOS inhibitor NG-nitro-L-arginine methyl ester (Fig. 2c).

### Paramylon upregulates TNF-α and IL-6

**Fig. 3:**
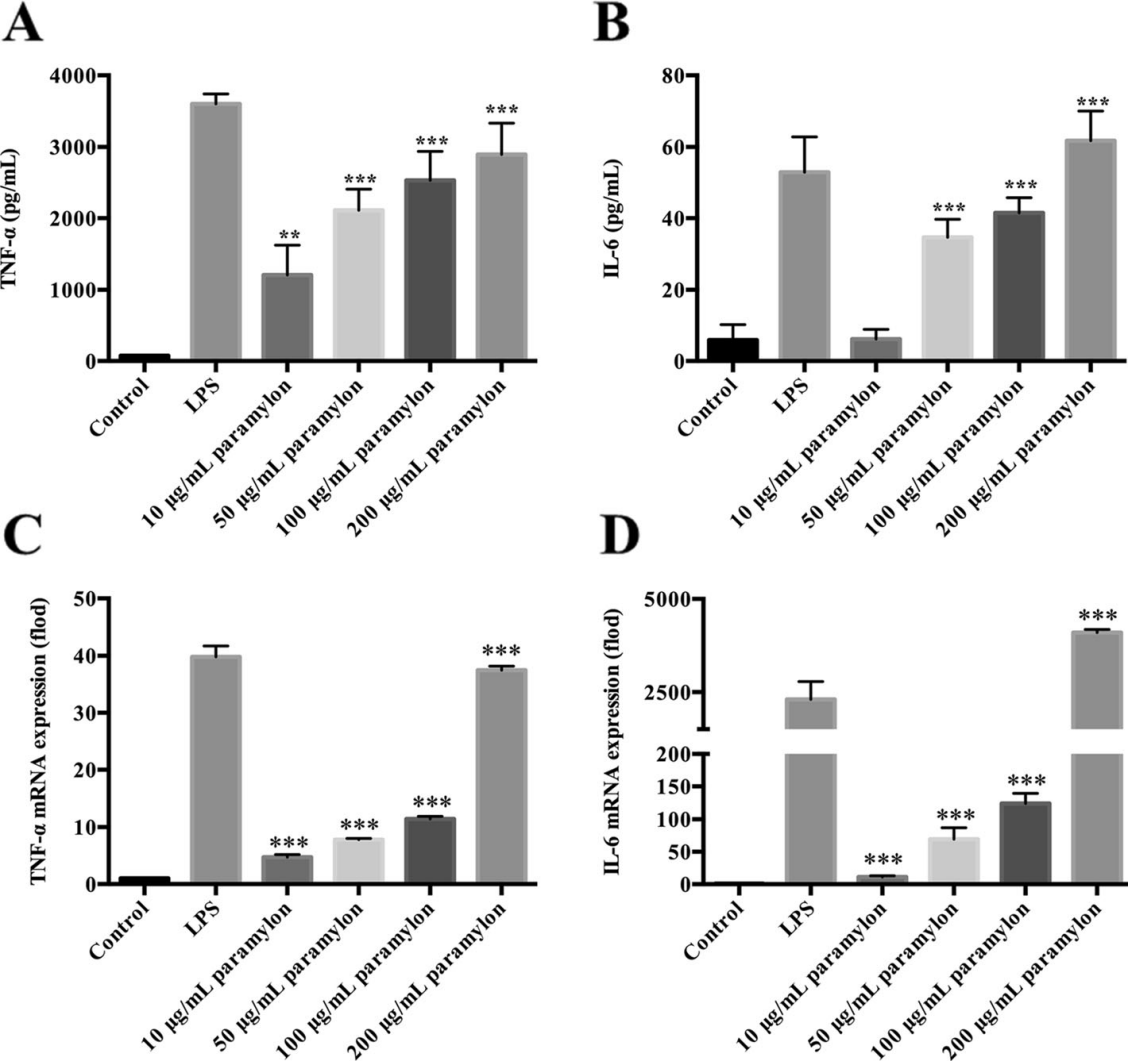
Paramylon induces TNF-α and IL-6 secretion. RAW264.7 cells were incubated with the different concentrations of paramylon shown for 12 h (for RNA test) or 24 h (for protein test). The production of TNF-α (**a**) and IL-6 (**b**) in the culture supernatant was detected by ELISA. The mRNA levels of the same genes were also analyzed by qRT-PCR (**c**, **d**). Representative results from three independent experiments are shown. * P < 0.05, ** P < 0.01, *** P < 0.001

Cytokines are an important part of innate immunity, as they form a hub for the body to exert immune function [25]. Activated macrophages secrete cytokines, such as TNF-α and IL-6, when the body is invaded by pathogenic microorganisms. We thus measured TNF-α and IL-6 levels, in paramylon-treated RAW264.7 macrophages, by ELISA. As shown in Figs. 3a and b, TNF-α and IL-6 production were concentration-dependently elevated upon paramylon treatment. qRT-PCR analysis demonstrated the same paramylon stimulation of TNF-α and IL-6 at the mRNA level (Fig. 3c, d).

### Paramylon activates NF-κB signaling pathway

**Fig. 4:**
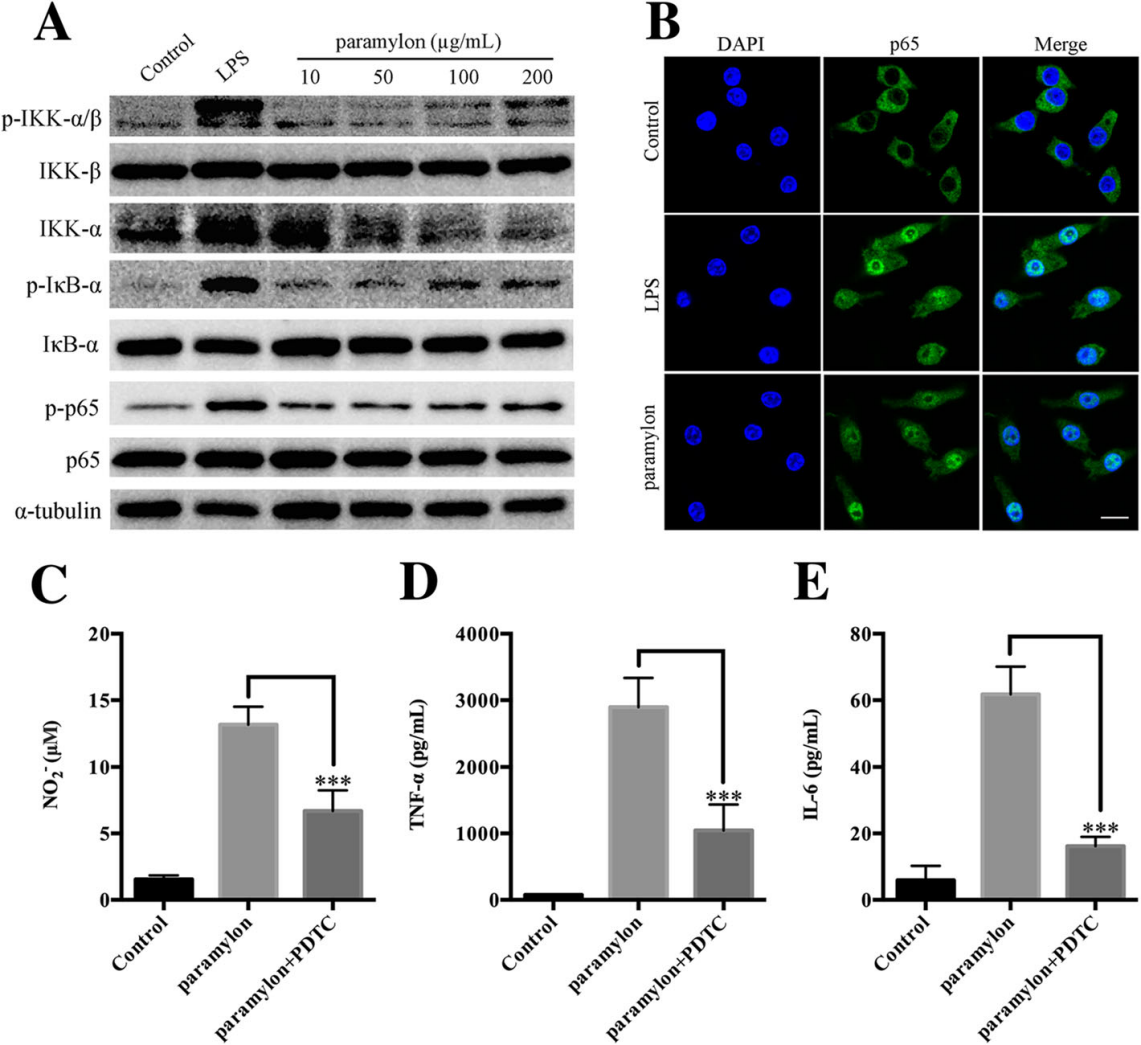
Paramylon activates NF-κB signaling. **a** The levels of p-IKK-α/β, IKK-β, IKK-α, phosphor-IκB-α (p-IκB-α), IκB-α, p-p65, p65 were detected in RAW264.7 cells by Western blot and normalized to α-tubulin. Lipopolysaccharide (LPS, 100 ng/mL) was used as a positive control. **b** Nuclear translocation of the NF-κB/p65 subunit was observed by confocal microscopy. DAPI was used to label the nucleus. Cells in 96-well plates (1 × 10^5^ cells/well) were incubated with 200 μg/mL paramylon with NF-κB signaling pathway inhibitor pyrrolidine dithiocarbamate (PDTC, 50 μM) to measure NO (**c**), TNF-α (**d**) and IL-6 (**e**) levels in the culture medium. Representative results from three independent experiments are shown, *** P < 0.001

The NF-κB signaling pathway triggers, and mediates the development of, various inflammatory responses [26]. We stimulated RAW264.7 macrophages with paramylon, and assayed for activation of the NF-κB signaling pathway by Western blot. As shown in Fig. 4a, paramylon induced phosphorylated IKK, IκB-α, and p65 in a concentration-dependent manner, indicating that paramylon activated the NF-κB signaling pathway.

Nuclear translocation of NF-κB/p65 is also a key step in the activation of the NF-κB signaling pathway. Therefore, immunofluorescence analysis was performed to determine whether paramylon can promote nuclear translocation of the NF-κB/p65 subunit. Consistent with NF-κB activation, the p65 subunit was significantly translocated to the nucleus after 2 h of treatment with paramylon, at a similar level as the lipopolysaccharide positive control (Fig. 4b). Pyrrolidine dithiocarbamate, an inhibitor of the NF-κB signaling pathway, also suppressed the production of NO, TNF-α and IL-6 in paramylon-stimulated cells (Fig. 4c-e).

### Paramylon activates MAPK signaling

**Fig. 5:**
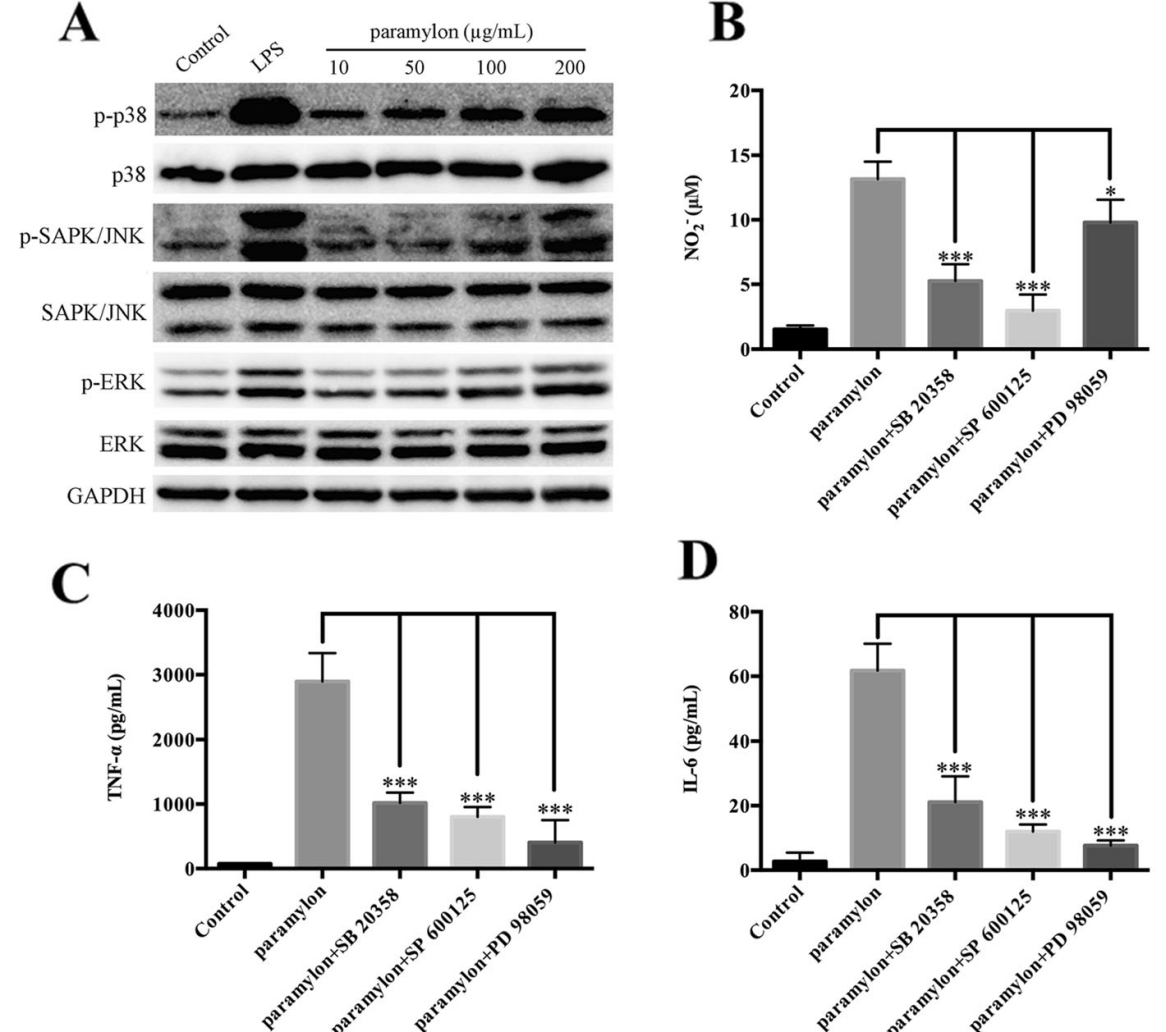
Activation of the MAPK signaling pathway by paramylon. **a** The levels of p-p38, p38, p-SAPK/JNK, SAPK/JNK, p-ERK, ERK were detected by Western blot analysis and normalized to GAPDH. Lipopolysaccharide (LPS, 100 ng/mL) was used as positive control. RAW264.7 cells in 96-well plates (1 × 10^5^ cells/well) were incubated with 200 μg/mL paramylon with, p38-inhibitor SB 20358 (10 μM), JNK inhibitor SP 600125 (10 μM) and ERK inhibitor PD 98059 (10 μM) to measure NO (**b**), TNF-α (**c**) and IL-6 (**d**) levels in the culture medium. Representative results from three independent experiments are shown, * P < 0.05, *** P < 0.001

MAPK is a signaling pathway regulated by protein phosphorylation and dephosphorylation, which in turn regulates a variety of physiological processes [27]. In the inflammatory response, the MAPK signaling pathway controls and regulates the expression of downstream genes such as iNOS, TNF-α, and IL-6 through further protein phosphorylation [4]. We thus used Western blot to determine the activation of the MAPK signaling pathway by paramylon. As shown in Fig. 5a, paramylon increased phosphorylated p38, JNK and ERK, in a concentration-dependent manner, suggesting that paramylon activates the MAPK signaling pathway.

In addition, we determined the effect of p38, JNK, and ERK inhibitors (SB20358, SP600125 and PD98059 respectively), on the stimulation of NO, TNF-α and IL-6 by paramylon. The effect of paramylon on all three cytokines was effectively suppressed by all three MAPK pathway inhibitors (Fig. 5b-d).

## Discussion

β-glucans are natural polysaccharides with immuno-stimulatory activity that form multi-component isoforms whose immunological activity depends on the molecular structure [4]. Paramylon is a mixed linear (unbranched) β-(1,3)-glucan polysaccharide polymer produced by *E. gracilis* [2]. Paramylon is insoluble in water and its molecular weight is estimated to be more than 500 kDa [28]. Sonication does not cause any cracking of paramylon, while alkali treatment can reduce the degree of polymerization of paramylon from ~ 3000 to ~ 70 and molecular weight from ~ 500 kDa to ~ 12 kDa [29].

Paramylon has a variety of biological activities, but little research has been done on its immunological activity. Recent studies have shown that paramylon activates lymphocytes by upregulating pro-inflammatory factors [30]. Russo et al. documented the β-glucan-stimulation products in human lymphocytes, and demonstrated that ultrasound- and alkalized-paramylon up-regulates pro-inflammatory cytokines. We investigated the effects of paramylon in mouse macrophages. It is clearly manifested in the paramylon increased transactivation of NF-κB visualized by immunofluorescence microscopy and detected the process of nuclei entering of NF-kB using the confocal microscope in this study [30]. However, the previous report did not have the false positive control (lipopolysaccharide-inhibitor polymyxin B, PMB) as we did in this study to get rid of the potential pollution of LPS in paramylon [30]. Kankkunen et al. demonstrated that β-glucan envelope-associated dectin-1 as the receptor and cytoplasmic NLRP3 inflammatory cytoplasmic cells were recognized by paramylon-activated dectin-1 and NLRP3 inflammatory bodies in human macrophages, respectively, resulting in human giant IL-1β gene up-regulation and IL-1β secretion in phagocytes [31]. Sonck et al. have tested effects of paramylon on porcine leukocytes and showed its significant activation effect on ROS production in neutrophils and monocytes [28].

In this study, we prepared ultrasound and alkalized paramylon from *E. gracilis*. Macrophages are phagocytic cells that play a major role in the host defense system by phagocytosis or by the production of NO and TNF-α to kill foreign infectious bacteria, agents and even tumor cells [14]. As a key messenger, NO plays a significant role in the immune response during pathogen infection [32]. Our results indicate that paramylon significantly induces NO production in RAW264.7 macrophages in a dose-dependent manner (Fig. 1c). At the same time the paramylon also upregulates iNOS RNA and protein in a dose-dependent manner (Fig. 2a, b). Similar trends in NO production and iNOS expression in human lymphocytes were observed with paramylon treatment [30]. L-NAME, an NOS inhibitor, inhibits paramylon-induced NO release in RAW264.7 macrophages (Fig. 2c), demonstrating that paramylon-stimulated NO production is achieved by iNOS.

It was well known that the pro-inflammatory cytokines secreted by immune cells play an important role in host defenses [14]. Paramylon extracted from *E. gracilis* has the ability to promote the production of TNF-α and IL-6 by human macrophages [30], and we confirmed the promotion of both mRNA and protein levels of cytokines in macrophages. Our study demonstrates that paramylon significantly induced TNF-α and IL-6 in RAW264.7 macrophages in a concentration-dependent manner (Fig. 4a, b). It further verifies that paramylon activates macrophages and enhances immune responses. In summary, paramylon is an effective macrophage activator.

We investigated the both NF-kB and MAPK pathways while only trans-activation of NF-kB was conducted in human cells [30]. We demonstrate that paramylon treatment also upregulates phosphorylation of IKK-β and IκB-α, resulting in increased phosphorylation of p65 (Fig. 4a). In resting cells, NF-κB migrates as a cell matrix p65-p50 dimer and binds to IκB. When exposure to pro-inflammatory stimuli such as LPS, TNF-α or IL-6, IκB rapidly phosphorylates and will be proteasomally degraded. The NF-κB p65-p50 dimer is then released and transferred to the nucleus where transcription of target genes would be induced, such as iNOS, TNF-α and IL-6 [33]. Our results demonstrate that paramylon stimulates RAW264.7 macrophages to induce phosphorylation of IκB-α resulting in phosphorylation and nuclear translocation of p65 (Fig. 4a, b). We speculate that paramylon acts as a potent macrophage activator that boosts the NF-κB signaling pathway. Our speculation was confirmed using NF-κB specific inhibitors. Paramylon-induced NO, IL-6 and TNF-α was significantly inhibited by NF-κB inhibitors (Fig. 4c-e). Thus, the supplementary and healthy food *Euglena* could be feed as immune activator.

The MAPK signaling pathway is composed of p38, JNK and ERK, as an important regulator in activated macrophages [30, 34]. Phosphorylation of p38, JNK, and ERK was dose-dependent in paramylon-treated RAW264.7 cells (Fig. 5a), confirming that paramylon-induced macrophage activation may be through the MAPK signaling pathway. Similarly, we speculate that paramylon acts as a potent macrophage boosts that activates the p38-MARK, JNK-MAPK and ERK-MAPK signaling pathways. Our propose was confirmed by the inhibitory action of p38, JNK and ERK-specific on paramylon-induced NO, IL-6 and TNF-α (Fig. 5b-d).

The mechanism of β-glucan action in the organism was thought mediated with several receptors, including scavenger receptors, complement receptor 3 (CR3), Toll-like receptors (TLR), lactosylceramide, and more recently, dectin-1 [31, 35, 36]. The recognition of β-glucans was originally thought to involve multiple interactions with these receptors, but dectin-1 has emerged as the primary receptor for this polysaccharide, at least in leukocytes [4, 37, 38]. β-glucans are potent immunomodulators with many potential applications. The discovery of dectin-1 has provided some fundamental insights into the molecular mechanisms underlying the activity of the polysaccharide, and has also extended our understanding of functioning of the innate immune system, but their mechanisms of action are largely unknown.

## Conclusion

**Fig. 6:**
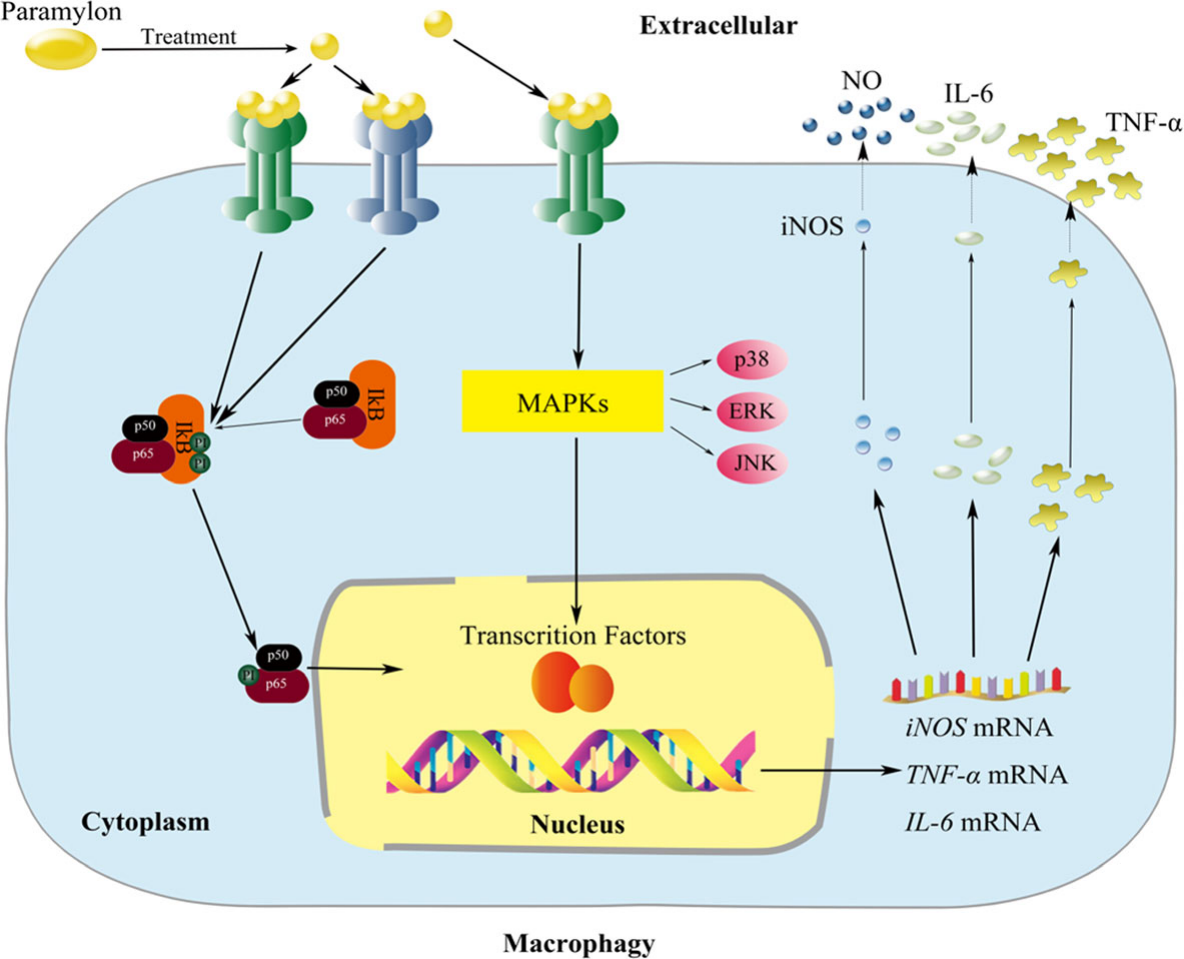
Immune activation of the paramylon extracted from *E. gracilis* in RAW264.7 cells. After being recognized by receptors on the membrane of macrophages, members of NF-κB and MAPK signaling pathways were phosphorylated and thus activated the related immune genes in the nucleus such as *iNOS*, *TNF-α* and *IL-6* which were translated into proteins to produce NO, TNF-α and IL-6 and exert immunomodulatory effects

In summary, our results indicate that sonicated and alkalized paramylon has immunological activation similar to LPS, and it is the first demonstration that paramylon from *E. gracilis* can dramatically activate the immune response in murine RAW264.7 cells. Paramylon induces the production of NO, TNF-α and IL-6 by activating NF-κB and MAPK signaling pathways (Fig. 6). Our findings provide important insights for the potential therapy potential of *E. gracilis* paramylon, which may be a useful resource for drug and health food development.

## Methods

### Algal strain and culture

*E. gracilis* CCAP 1224/5Z was obtained from CCAP (Culture Collection of Algae and Protozoa, UK) and maintained in medium (1.8 g/L NH_4_Cl, 0.6 g/L KH_2_PO_4_, 0.6 g/L MgSO_4_, 60 mg/L Urea, 0.02 g/L CaCl_2_, 0.48 mg/L Na_2_EDTA, 2 mg/L Fe_2_ (SO_4_)_3_, 10 ml EtOH, 60 μL HCl, 0.01 mg/L Vb_1_, 0.0005 mg/L Vb_12_, 20 mg/L CuSO_4_·5H_2_O, 0.4 g/L ZnSO_4_·7H_2_O, 1.3 g/L Co (NH_3_)·H_2_O, 1.6 g/L MnCl_2_·4H_2_O). Cells were grown at 22 °C photo-incubator under 100 μmol/m^2^/s with hand shaking twice every day, and cells at the exponential growth period were harvested for further tests.

## Materials

Lipopolysaccharide, polymyxin B, NG-nitro-L-arginine methyl ester and pyrrolidine dithiocarbamate were purchased from Sigma-Aldrich (St. Louis, MO, USA). The inhibitors: SB 20358, SP 600125 and PD 98059, were purchased from Selleck (Shanghai, China). The Cell Counting Kit (CCK)-8 and Radioimmunoprecipitation Assay buffer were purchased from Beyotime (Jiangsu, China). Antibodies (against iNOS, IκB-α, phosphor-IκB-α, p65, p-p65, p38, p-p38, JNK, p-JNK, ERK, p-ERK) and horseradish peroxidase conjugated secondary antibody were obtained from Cell Signaling Technology (Beverly, MA, USA). Antibodies against alpha-tubulin and GAPDH were obtained from Proteintech (Hubei, China).

### Preparation of paramylon

Five-day-old *E. gracilis* were collected by centrifugation at 5500 g for 5 min and washed twice with deionized water. Sonication was then used to break up the cells. Paramylon is insoluble, so to remove lipids and proteins, the sonicate was solubilized in a 1% (w/v) sodium dodecyl sulfate (SDS) solution at 95 °C dry bath for 1 h. The crude paramylon was then precipitated by centrifugation at 5000 g for 5 min and treated at 50 °C for 30 min with a 0.1% SDS solution. Further centrifugation was carried out at 3000 g for 5 min, and the pellet was washed separately with water, acetone, diethyl ether and centrifuged [39]. The pellet was then dissolved in 0.5 M NaOH, two volumes of cold 98% ethanol were added to the pellet and the mixture was centrifuged at 12,000 g for 10 min at 4 °C. The ethanol precipitation was repeated once and the final pellet was suspended in 30 mL of deionized water and the pH value was adjusted to 7.0 with 2 M HCl. Paramylon suspension is ultrasonically treated on the ice for 12 min (12 48-s ultrasonic treatment cycles with 12-s cycle intervals). One mL aliquots were made and stored at − 20 °C.

### Cell culture and processing

RAW264.7 macrophages were cultured in Dulbecco’s modified Eagle’s medium (DMEM) containing 10% (v/v) fetal bovine serum (FBS) and 1% Antibiotic-Antimycotic (Cat No. 15240062, Gibco, Grand Island, NY, USA) at 37 °C, 5% CO_2_. The cells were seeded into 96-well (1 × 10^5^ cells/well) or 6-well plates (1 × 10^6^ cells/well) for 4 h, and then incubated with 10, 50, 100 or 200 μg/mL paramylon or 100 ng/mL lipopolysaccharide (positive controls) for 30 min or 24 h.

### Cell viability assay

After treatment with paramylon or lipopolysaccharide for 24 h, 10 μL of cell counting kit reagents was added to the cells. After 1 h of incubation, the absorbance of each well was measured at A450 nm using a SpectraMax microplate reader (SpectraMax 190, Molecular Devices, USA).

### NO assay

After treatment with paramylon or lipopolysaccharide for 24 h, 50 μL supernatant was mixed with 50 μL sulfonamide reagent (SUL, Sangon Biotech, Shanghai) and then 50 μL naphthalene ethylenediamine reagent (NED, Sangon Biotech, Shanghai) was added into mixture. After a 5 min incubation, the absorbance was detected at 545 nm, and the NO_2_^−^ content was calculated according to the standard curve of NaNO_2_ [40]. In inhibition experiment, RAW264.7 cells were pre-treated with polymyxin B for 2 h, and then co-cultured with 100 ng/mL LPS or 200 μg/mL paramylon 24 h.

### Cytokine assay

After treatment with paramylon or lipopolysaccharide, cellular TNF-α and IL-6 levels were measured using an ELISA kit (Neobioscience, Guangdong, China) according to the manufacturer’s protocol. In the inhibition experiment, adherent cells were pretreated with the inhibitor for 2 h and then co-cultured with 200 μg/mL paramylon for 24 h.

### RNA isolation and quantitative real-time polymerase chain reaction(qRT-PCR)

qRT-PCR was used to quantify gene expression of IL-6, TNF-α and iNOS, and GAPDH was used as reference housekeeping gene. After treating RAW264.7 cells with paramylon or lipopolysaccharide for 12 h, total RNA was isolated, with an RNeasy Mini Kit (Qiagen, Hilden, Germany), and reversed transcribed, using the iScript™ cDNA Synthesis Kit (Bio-Rad, Hercules, California, CA). qRT-PCR was performed with SoFast™ EvaGreen® Supermix (Bio-Rad, Hercules, California, CA) in the StepOne Plus™ Real-Time PCR System (ABI Applied Biosystems, Foster City, CA). Gene primers were designed using Beacon Designer software (Premier Biosoft, Palo Alto, California, USA) (Table 1). Samples were assayed in triplicates and gene expression was calculated using the 2^-ΔΔCt^ relative quantification method [41]. The amount of each transcript was normalized to the GAPDH transcript in the same cDNA sample.

**Table 1:** Primers used for quantitative Real-time Polymerase Chain Reaction

Primers	Forward	Reverse
*iNOS*	5′-CAGCTCAAGAGCCAGAAACG-3′	5′-TTACTCAGTGCCAGAAGCTG-3′
*IL-6*	5′-CCAATTTCCAATGCTCTCCT-3′	5′-ACCACAGTGAGGAATGTCCA-3′
*IL-1β*	5′-CTTTGAAGAAGAGCCCATCC-3′	5′-TTTGTCGTTGCTTGGTTCTC-3′
*TNF-α*	5′-GACCCTCACACTCAGATCATCTTCT-3′	5′-CCTCCACTTGGTGGTTTGCT-3′
*GAPDH*	5′-ATGCCTCCTGCACCACCA-3′	5′-CCATCACGCCACAGTTTCC-3′

### Western blot analysis

After 24 h of treatment with different concentrations of paramylon or lipopolysaccharide, the total cellular protein (20 μg) in each sample was separated by 8% or 12% SDS-PAGE and transferred to a PVDF membrane. After blocking with 5% (w/v) fat-free milk, the membrane was incubated with the primary antibody (1: 1000 to dilute) at 4 °C overnight, and rinsed thoroughly with the secondary antibody (1: 5000 to dilute) for 1 h at room temperature. The membrane was then detected using an electroluminescent kit (Thermo Fisher Scientific, Waltham, MA, USA).

### Immunocytochemistry of NF-kB

RAW264.7 cells were seeded on sterile glass coverslips in 6-well plates (1 × 10^6^ cells/well) and treated with 200 μg/mL paramylon or 100 ng/mL lipopolysaccharide for 2 h. The cells on the coverslips were fixed immediately in 4% (v/v) formaldehyde at RT for 30 min. After being permeabilized with 0.2% (v/v) Triton X-100 in PBS for 10 min, cells were blocked with 10% (w/v) goat serum in PBS, at 37 °C for 1 h. The cells were then incubated with an NF-κB/p65 primary antibody at 4 °C overnight. After three washes in cold PBS, the cells were incubated with an Alexa Fluor 596-conjugated secondary antibody and DAPI at RT for 2 h. The nuclear translocation of the NF-κB/p65 subunit was observed by confocal microscopy (Carl Zeiss Jena Gmbh, Jena, Germany) [42].

### Statistical analysis

All the data were indicated by mean ± standard deviations (mean ± SD) and statistically analyzed by two-tailed Student’s t-test in GraphPad Prism 7 (GraphPad Software, Inc., La Jolla, CA, USA). *p* value less than 0.01 (*p* < 0.01) was considered extremely significantly different, *p* < 0.05 indicating statistically different, and *p* value more than 0.05 (*p* > 0.05) was not significant.

## Data Availability

The datasets used and/or analyzed during the current study are available from the corresponding author on reasonable request. All data generated or analyzed during this study are included in this published article.

## Abbreviations

ALRs: AIM-2-like receptors
CCK: Cell Counting Kit
CCAP: Culture Collection of Algae and Protozoa
CLRs: C-type lectin receptors
DMEM: Dulbecco’s modified Eagle’s medium
FBS: fetal bovine serum
IL-6: interleukin-6
IFN: interferon
iNOS: nitric oxide synthase
MAPK: mitogen-activated protein kinase
NED: naphthalene ethylenediamine reagent
NF-κB: nuclear factor-κB
NLRs: Nod-like receptors
NO: nitric oxide
qRT-PCR: quantitative Real-time Polymerase Chain Reaction
RLRs: RIG-I-like receptors
ROS: reactive oxygen species
SDS: sodium dodecyl sulfate
SUL: sulfonamide reagent
TLRs: Toll-like receptors
TNF-α: tumor necrosis factor-α

## References

[1] Barsanti L, Passarelli V, Evangelista V, Frassanito AM, Gualtieri P (2011). Chemistry, physico-chemistry and applications linked to biological activities of β-glucans. Nat Prod Rep.

[2] Barsanti L, Vismara R, Passarelli V, Gualtieri P (2001). Paramylon (β-1, 3-glucan) content in wild type and WZSL mutant of Euglena gracilis. Effects of growth conditions. J Appl Phycol.

[3] Monfils A, Triemer R, Bellairs E (2011). Characterization of paramylon morphological diversity in photosynthetic euglenoids (Euglenales, Euglenophyta). Phycologia..

[4] Tsoni SV, Brown GD (2008). β-Glucans and Dectin-1. Ann N Y Acad Sci.

[5] Nakashima A, Suzuki K, Asayama Y, Konno M, Saito K, Yamazaki N, Takimoto H (2017). Oral administration of Euglena gracilis Z and its carbohydrate storage substance provides survival protection against influenza virus infection in mice. Biochem Biophys Res Commun.

[6] Quesada LA, Lustig ES, Marechal LR, Belocopitow E (1976). Antitumor activity of paramylon on sarcoma-180 in mice. Jpn J Cancer Res.

[7] Watanabe T, Shimada R, Matsuyama A, Yuasa M, Sawamura H, Yoshida E, Suzuki K (2013). Antitumor activity of the β-glucan paramylon from Euglena against preneoplastic colonic aberrant crypt foci in mice. Food Funct.

[8] MacMicking J, Xie QW, Nathan C (1997). Nitric oxide and macrophage function. Annu Rev Immunol.

[9] Torreilles J, Guérin MC, Roch P (1997). Peroxidase-release associated with phagocytosis in Mytilus galloprovincialis haemocytes. Dev Comp Immunol.

[10] Costa MM, Novoa B, Figueras A (2008). Influence of β-glucans on the immune responses of carpet shell clam (Ruditapes decussatus) and Mediterranean mussel (Mytilus galloprovincialis). Fish Shellfish Immun.

[11] Costa MM, Prado-Alvarez M, Gestal C, Li H, Roch P, Novoa B, Figueras A (2009). Functional and molecular immune response of Mediterranean mussel (Mytilus galloprovincialis) haemocytes against pathogen-associated molecular patterns and bacteria. Fish Shellfish Immun.

[12] Gissibl A, Care A, Parker LM, Iqbal S, Hobba G, Nevalainen H, Sunna A (2018). Microwave pretreatment of paramylon enhances the enzymatic production of soluble β-1, 3-glucans with immunostimulatory activity. Carbohydr Polym.

[13] Yang Y, Zhao X, Li J, Jiang H, Shan X, Wang Y, Ma W, Hao J, Yu G (2018). A β-glucan from Durvillaea Antarctica has immunomodulatory effects on RAW264. 7 macrophages via toll-like receptor 4. Carbohydr Polym.

[14] Akira S, Uematsu S, Takeuchi O (2006). Pathogen recognition and innate immunity. Cell..

[15] Classen A, Lloberas J, Celada A. Macrophage activation: classical vs. alternative. In: Macrophages and Dendritic Cells. Springer; 2009: 29–43. 10.1007/978-1-59745-396-7_3 19347309

[16] Kohchi C, Inagawa H, Nishizawa T, Soma GIJAR (2009). ROS and Innate immunity. Anticancer Res.

[17] Guha M, Mackman N (2001). LPS induction of gene expression in human monocytes. Cell Signal.

[18] Zhang G, Ghosh S (2001). Toll-like receptor-mediated NF-kappaB activation: a phylogenetically conserved paradigm in innate immunity. J Clin Invest.

[19] Wu J, Zhou J, Chen X, Fortenbery N, Eksioglu EA, Wei S, Dong J (2012). Attenuation of LPS-induced inflammation by ICT, a derivate of icariin, via inhibition of the CD14/TLR4 signaling pathway in human monocytes. Int Immunopharmacol.

[20] Cui J, Chen Y, Wang HY, Wang RF (2014). Mechanisms and pathways of innate immune activation and regulation in health and cancer. Hum Vaccin Immunother.

[21] Phillips FC, Jensen GS, Showman L, Tonda R, Horst G, Levine R (2019). Particulate and solubilized beta-glucan and non-beta-glucan fractions of Euglena gracilis induce pro-and anti-inflammatory innate immune cell responses and exhibit antioxidant properties. J Inflamm Res.

[22] Savva A, Roger T (2013). Targeting toll-like receptors: promising therapeutic strategies for the management of sepsis-associated pathology and infectious diseases. Front Immunol.

[23] Kumar H, Kawai T, Akira S (2011). Pathogen recognition by the innate immune system. Int Rev Immunol.

[24] Stuehr DJ, Gross SS, Sakuma I, Levi R, Nathan CF (1989). Activated murine macrophages secrete a metabolite of arginine with the bioactivity of endothelium-derived relaxing factor and the chemical reactivity of nitric oxide. J Exp Med.

[25] Savage ND, de Boer T, Walburg KV, Joosten SA, van Meijgaarden K, Geluk A, Ottenhoff TH (2008). Human anti-inflammatory macrophages induce Foxp3+ GITR+ CD25+ regulatory T cells, which suppress via membrane-bound TGFβ-1. J Immunol.

[26] Kang SR, Han DY, Park KI, Park HS, Cho YB, Lee HJ, Lee WS, Ryu CH, Ha YL, Lee DH. Suppressive effect on lipopolysaccharide-induced proinflammatory mediators by *Citrus aurantium* L. in macrophage RAW 264.7 cells via NF-B signal pathway. Evidence-Based Evid-Based Compl Alt. 2011; 2011. 10.1155/2011/248592 PMC2952293 20953420

[27] Kim JA, Ahn BN, Kong CS, Kim SK (2011). Anti-inflammatory action of sulfated glucosamine on cytokine regulation in LPS-activated PMA-differentiated THP-1 macrophages. Inflamm Res.

[28] Sonck E, Stuyven E, Goddeeris B, Cox E (2010). The effect of β-glucans on porcine leukocytes. Vet Immunol Immunopathol.

[29] Tamura N, Wada M, Isogai A (2009). TEMPO-mediated oxidation of (1, 3)-β-d-glucans. Carbohydr Polym.

[30] Russo R, Barsanti L, Evangelista V, Frassanito AM, Longo V, Pucci L, Penno G, Gualtieri P (2017). Euglena gracilis paramylon activates human lymphocytes by upregulating pro-inflammatory factors. Food Sci Nutr.

[31] Kankkunen P, Teirila L, Rintahaka J, Alenius H, Wolff H, Matikainen S (2010). (1,3)- β-glucans activate both dectin-1 and NLRP3 inflammasome in human macrophages. J Immunol.

[32] Bi D, Yu B, Han Q, Lu J, White WL, Lai Q, Cai N, Luo W, Gu L, Li S (2018). Immune activation of RAW264.7 macrophages by low molecular weight Fucoidan extracted from New Zealand Undaria pinnatifida. J Agric Food Chem.

[33] Tak PP, Firestein GS (2001). NF-κB: a key role in inflammatory diseases. J Clin Invest.

[34] Liu Y, Shepherd EG, Nelin LD (2007). MAPK phosphatases—regulating the immune response. Nat Rev Immunol.

[35] Goodridge HS, Wolf AJ, Underhill DM (2009). β-Glucan recognition by the innate immune system. Immunol Rev.

[36] Baert K, Sonck E, Goddeeris BM, Devriendt B, Cox E (2015). Cell type-specific differences in β-glucan recognition and signalling in porcine innate immune cells. Dev Comp Immunol.

[37] Noss I, Doekes G, Thorne PS, Heederik DJ, Wouters IM (2013). Comparison of the potency of a variety of β-glucans to induce cytokine production in human whole blood. Innate Immun.

[38] Legentil L, Paris F, Ballet C, Trouvelot S, Daire X, Vetvicka V, Ferrières V (2015). Molecular interactions of β-(1, 3)-glucans with their receptors. Molecules..

[39] Sugiyama A, Suzuki K, Mitra S, Arashida R, Yoshida E, Nakano R, Yabuta Y, Takeuchi T (2009). Hepatoprotective effects of paramylon, a β-(1, 3)-D-glucan isolated from Euglena gracilis Z, on acute liver injury induced by carbon tetrachloride in rats. J Vet Med Sci.

[40] Fang W, Bi D, Zheng R, Cai N, Xu H, Zhou R, Lu J, Wan M, Xu X (2017). Identification and activation of TLR4-mediated signalling pathways by alginate-derived guluronate oligosaccharide in RAW264. 7 macrophages. Sci Rep.

[41] Kankkunen P, Rintahaka J, Aalto A, Leino M, Majuri M-L, Alenius H, Wolff H, Matikainen S (2009). Trichothecene mycotoxins activate inflammatory response in human macrophages. J Immunol.

[42] Bi D, Lai Q, Cai N, Li T, Zhang Y, Han Q, Peng Y, Xu H, Lu J, Bao W (2018). Elucidation of the molecular-mechanisms and in vivo evaluation of the anti-inflammatory effect of alginate-derived Seleno-polymannuronate. J Agric Food Chem.

